# Evaluation of treatment planning feasibility of a positron emission tomography (PET)–Linac system for spine stereotactic body radiotherapy (SBRT)

**DOI:** 10.1002/acm2.70307

**Published:** 2025-11-29

**Authors:** Haozhao Zhang, Shunyu Yan, Hao Peng, Thomas Banks, Rameshwar Prasad, Yang Kyun Park, Andrew Godley, Bin Cai, Chenyang Shen

**Affiliations:** ^1^ Department of Radiation Oncology The University of Texas Southwestern Medical Center Dallas Texas USA; ^2^ Medical Artificial Intelligence and Automation Laboratory The University of Texas Southwestern Medical Center Dallas Texas USA

**Keywords:** planning, RefleXion, spine SBRT

## Abstract

**Background and Purpose:**

The RefleXion X1 (RefleXion Medical, Inc., Hayward, CA) is a unique radiotherapy platform integrating kVCT and PET as on‐board imaging guidance. This study aims to evaluate the feasibility and treatment planning quality of RefleXion X1 treatment planning system (TPS) for spinal stereotactic body radiation therapy (SBRT).

**Methods:**

This retrospective study analyzed treatment plans of 10 different patient cases, including five single‐dose level SBRT cases and five simultaneously integrated boost (SIB) SBRT cases. All plans were created using the X1 TPS with a 10 mm jaw size and evaluated against institutional planning guidelines to ensure clinical acceptability. To assess planning quality, the X1‐generated plans were compared to clinically treated plans. Key metrics such as planning target volume (PTV) coverage, maximum point dose (measured as maximal dose to any volume of 0.035cc), conformity index (CI), and organs‐at‐risk (OARs) constraints were evaluated. Statistical analysis was performed using the Wilcoxon signed‐rank test, with significance defined as *p* < 0.05.

**Results:**

All RefleXion X1 plans met our institutional planning guidelines for spinal SBRT and were deemed clinically acceptable. No statistically significant differences were observed in all PTV metrics, including $D_{\max}$ (p=0.70), $D_{\mathrm{mean}}$ (p=0.77), and $D_{\min}$ (p=0.23). Meanwhile, X1 plans demonstrated slightly higher average maximum cauda equina doses (X1: 12.87 Gy, clinically treated: 11.57 Gy, p=0.05), while maintaining comparable dose sparing for other OARs. The average CI values for X1 plans and clinically treated plans were 1.19 and 1.18, respectively (p=0.67), demonstrating similar dose conformity. Additionally, homogeneity index (HI) and gradient index (GI) values also showed no significant differences, further supporting the comparable performance of X1 plans.

**Conclusions:**

The RefleXion X1 system can generate clinically acceptable treatment plans for complex spinal SBRT, with plan quality comparable to that of clinically treated plans at our institution.

## 1 INTRODUCTION

With the recent advancements in radiotherapy treatment planning and delivery, spine stereotactic body radiotherapy (SBRT)^1^ has become feasible and emerged as an effective treatment modality for spinal metastases.^2^, ^3^, ^4^, ^5^ It requires a highly accurate linear accelerator (LINAC) and a powerful treatment planning system (TPS) to design and deliver high dose per fraction to the tumor while minimizing radiation exposure to adjacent organs‐at‐risk (OARs). Given different clinical goals and practical considerations for different patients, single‐dose level SBRT treatments or simultaneously integrated boost (SIB) might be prescribed.^4^, ^5^, ^6^ Compared to single‐dose level SBRT, the SIB approach enables dose escalation to specific tumor regions for better tumor control probability but imposes additional complexity to the treatment planning process^4^, ^5^ Among various anatomical sites where SBRT is applied, spinal SBRT presents particularly unique challenges that make it an ideal testbed for evaluating advanced treatment planning systems due to the spine's complex anatomy, irregular target shapes, and proximity to critical neural structures requiring exceptionally steep dose gradients.

Recently, the RefleXion X1 system (RefleXion Medical, Inc., Hayward, CA) was introduced into clinical practice at our institution, offering a novel treatment option with on‐board kVCT and PET as imaging guidance capabilities. Previous studies have established the technical feasibility of the RefleXion X1 TPS for various treatment sites, including brain tumors and thoracic lesions,^7^, ^8^, ^9^ demonstrating comparable plan quality to conventional TPS for intensity modulated radiation therapy (IMRT) and SBRT applications. However, spinal SBRT presents unique technical challenges that remain unresolved for the X1 TPS: (1) the complex anatomical geometry with irregular target shapes; (2) the requirement for exceptionally steep dose gradients to deliver high doses per fraction (typically 15–24 Gy) while respecting strict dose constraints for abutting critical structures, for example, spinal cord; and (3) the planning complexity introduced by SIB approaches presenting additional optimization challenges for TPS compared to conventional two‐step sequential boost methods, as it requires simultaneous optimization of multiple dose levels within a single treatment plan while maintaining dose conformity and critical structure sparing. The sequential boost method allows separate optimization of different dose levels with additional degrees of freedom, thus posing fewer challenges to the optimization capability. To comprehensively evaluate the optimization limits of the RefleXion TPS, we chose to focus on the more challenging SIB approach, as successful SIB planning would demonstrate the system's capability to handle the most demanding optimization scenarios. Therefore, this study systematically evaluates the dosimetric quality and clinical acceptability of both single‐dose level and SIB spinal SBRT plans generated using the RefleXion X1 TPS with simulation CT images acquired on our clinical CT simulator and compares the plan quality against clinically treated plans generated using conventional TPS at our institution.

## 2 METHODS AND MATERIALS

### 2.1 Data collection

A total of 10 spine SBRT patients were retrospectively collected, including five patient cases treated with single‐dose level SBRT and another five treated with SIB. The lesions were located in either thoracic (6 cases) or lumbar spine (4 cases). All patients were simulated in the head‐first supine (HFS) position using a Philips Brilliance CT Big Bore scanner (Philips Medical Systems, Cleveland, OH, USA), with uniform slice thickness of 2 mm (axial plane matrix size: 512 × 512, tube current: 208 mA, tube voltage: 120kVp). Planning CT images were co‐registered with T1‐ and T2‐weighted magnetic resonance imaging (MRI), which were acquired with the patient positioned on a flat couch but without immobilization. These co‐registered images were used for target and spinal cord delineation. The clinical target volume (CTV) was delineated by experienced radiation oncologists following the recommendations of the International Spine Research Consortium.^10^ The PTV was created by applying a 2 mm isotropic expansion to the CTV. For SIB cases, PTV delineation for lower dose level followed the same methodology as single‐dose level plans. Additionally, a PTV‐Boost was manually delineated based on the gross tumor volume (GTV), using co‐registered CT, MRI, or PET‐CT images if available. Relevant OARs, including the spinal cord, esophagus, bowel, heart, liver, and kidneys, were also contoured and incorporated into a combined RT structure set. These paired structure sets and simulation CTs were used to generate RefleXion X1 plans and clinical plans.

### 2.2 Clinical treatment planning and delivery

The clinically treated plans for the 10 cases involved in this study were previously planned to use volumetric arc therapy (VMAT) with either 6MV flattening‐filter‐free (FFF) beams or 10MV FFF beams using the Eclipse TPS (version 16.1, Varian Medical Systems, Palo Alto, CA). Detailed characteristics of each plan are summarized in Table 1. Dose calculations were performed using Acuros XB (AXB) algorithms^11^ with calculation grid sizes of 1.25 mm. Optimization was conducted using the Photon Optimizer (PO) version 16.1. Each plan was carefully tailored to meet the clinical protocol at our hospital, which was developed based on NRG Oncology and RTOG guidelines, ensuring adequate target coverage and OAR sparing. All clinically treated plans were designed for two advanced LINACs at our institution, that is, the Varian TrueBeam (Varian Medical Systems, Palo Alto, CA) and Elekta Versa HD (Elekta AB, Stockholm, Sweden), respectively.

**TABLE 1:** Characteristics of selected clinically treated plans.

Plan	Treated level	Plan type	Prescription (Gy) /fractionation	Target Volume (cc)	Length of target (cm)	Algorithm	Beam energy (MV)	VMAT arc count	Machine
**1**	L3	*SDL	24/2	37.6	3.5	AXB	10X‐FFF	2	Versa HD
**2**	T6‐8	*SDL	40/5	139.8	5.4	AXB	6X‐FFF	2	TrueBeam
**3**	T10	*SDL	24/2	31.6	2.2	AXB	6X‐FFF	3	TrueBeam
**4**	L1‐2	*SDL	35/5	168.1	7.2	AXB	10X‐FFF	2	TrueBeam
**5**	T4	*SDL	35/5	26.0	3.4	AXB	10X‐FFF	2	TrueBeam
**6**	T12	SIB	40/5, 30/5	44.7, 5.8	3.2	AXB	6X‐FFF	3	TrueBeam
**7**	T9‐11	SIB	14/1, 20/1	69.6, 9.1	8.1	AXB	10X‐FFF	3	TrueBeam
**8**	L4	SIB	14/1, 20/1	50.4, 6.2	2.8	AXB	10X‐FFF	2	Versa HD
**9**	L3	SIB	14/1, 20/1	38.7, 12.8	2.9	AXB	10X‐FFF	3	TrueBeam
**10**	T8‐10	SIB	14/1, 20/1	100.9, 9.8	7.7	AXB	10X‐FFF	3	TrueBeam

*Note*: ^*^indicates that SDL refers to single‐dose level plans.

### 2.3 RefleXion X1 system and treatment planning

The RefleXion X1 system features a 6MV FFF LINAC mounted on a fast‐rotating ring gantry (60 rotations per minute in treatment), capable of delivering modulated beamlets at 50 gantry angles in each full rotation. The system leverages a nominal dose rate of 1000MU/min and uses jaw size of 10 mm or 20 mm and 64 binary multi‐leaf collimators (MLCs) (each with 6.25 mm in width) to modulate the treatment beam. The maximum width of field is 40 cm with a fixed 85 cm source‐to‐axis distance (SAD). Treatment on the X1 system is delivered in either single‐pass or four‐pass modes, with the couch moving at a discrete interval of 2.1 mm until the whole target volume is covered. A “pass” refers to the movement of the treatment couch in the IEC‐Y direction (head‐to‐toe axis), where the target volume moves through the treatment plane once. In four‐pass mode, the treatment couch moves in a back‐and‐forth fashion through the IEC‐Y axis, allowing each target volume to pass through the treatment plane four times. The four‐pass mode provides improved dose modulation capabilities and enhanced conformality compared to single‐pass delivery, which is particularly beneficial for complex targets requiring steep dose gradients. For this study, four‐pass treatment mode was primarily considered based on the vendor's recommendation.^7^, ^12^

Plans on the X1 TPS (v2.1.29) were generated using a jaw size of 10 mm. Optimization was performed using accelerated proximal gradient descent based on the Fast Iterative Shrinkage‐Thresholding Algorithm (FISTA)^13^ and dose calculations were conducted with the collapsed cone convolution superposition (CCCS) algorithm^12^, ^14^ using a calculation grid size of 2.1 mm^7^ A default smoothing parameter of 10 was used for all plans, which was used to limit the number of MLC transitions in the optimized plan.^7^ All plans were optimized to meet our institutional planning guidelines (partial important constraints shown in Table 2) for OARs with prescription dose (Rx) to cover at least 95% of PTV.^15^

**TABLE 2:** Institutional dose constraints for spinal cord and cauda equina employed in this study.

	*Fractionations*		
Target	1	2	5
**Spinal cord**	$V_{10\;\mathrm{Gy}}<0.35\mathrm{cc}$	$V_{13\;\mathrm{Gy}}<0.35\mathrm{cc}$	$V_{22\;\mathrm{Gy}}<0.35\mathrm{cc}$
	$D_{\max}<14\;\mathrm{Gy}$	$D_{\max}<18.3\;\mathrm{Gy}$	$D_{\max}<28\;\mathrm{Gy}$
**Cauda equina**	$V_{14\;\mathrm{Gy}}<5\mathrm{cc}$	$V_{18\;\mathrm{Gy}}<5\mathrm{cc}$	$V_{30\;\mathrm{Gy}}<5\mathrm{cc}$
	$D_{\max}<16\;\mathrm{Gy}$	$D_{\max}<20.8\;\mathrm{Gy}$	$D_{\max}<31.5\;\mathrm{Gy}$

### 2.4 Plan evaluation and comparison

For plan quality evaluation and comparison, the dose covering 98% of all PTVs (PTV $D_{98\%}$) and 2% of the PTV of highest dose level (PTV $D_{2\%}$) were collected for all plans along with maximum point dose ($D_{\max}$), minimum point dose ($D_{\min}$), and mean dose ($D_{\mathrm{mean}}$). The point dose was defined as the maximal doses delivered to any volume of 0.035cc. Conformality index (CI) was calculated as the ratio between the 100% isodose volume (V_RX_) and the PTV: $\mathrm{CI}\;=\frac{V_{\mathrm{RX}}}{V_{\mathrm{PTV}}}$.^16^ Homogeneity Index (HI) was calculated as the ratio of the difference between the maximum and minimum dose to Rx: $\mathrm{HI}\;=\frac{D_{\max-}D_{\min}}{\mathrm{Rx}}$.^17^ Gradient Index (GI) was computed as the ratio between the volume receiving 50% of the prescribed dose (V_50%RX_) and the volume receiving 100% of the prescribed dose (V_RX_): $\mathrm{GI}\;=\frac{V_{50\%\mathrm{RX}}}{V_{\mathrm{RX}}}$.^18^ Comparisons between the RefleXion X1 plans and the corresponding clinically treated plans were conducted using Wilcoxon signed‐rank test with a *p*‐value threshold of < 0.05.

## 3 RESULTS

**FIGURE 1:**
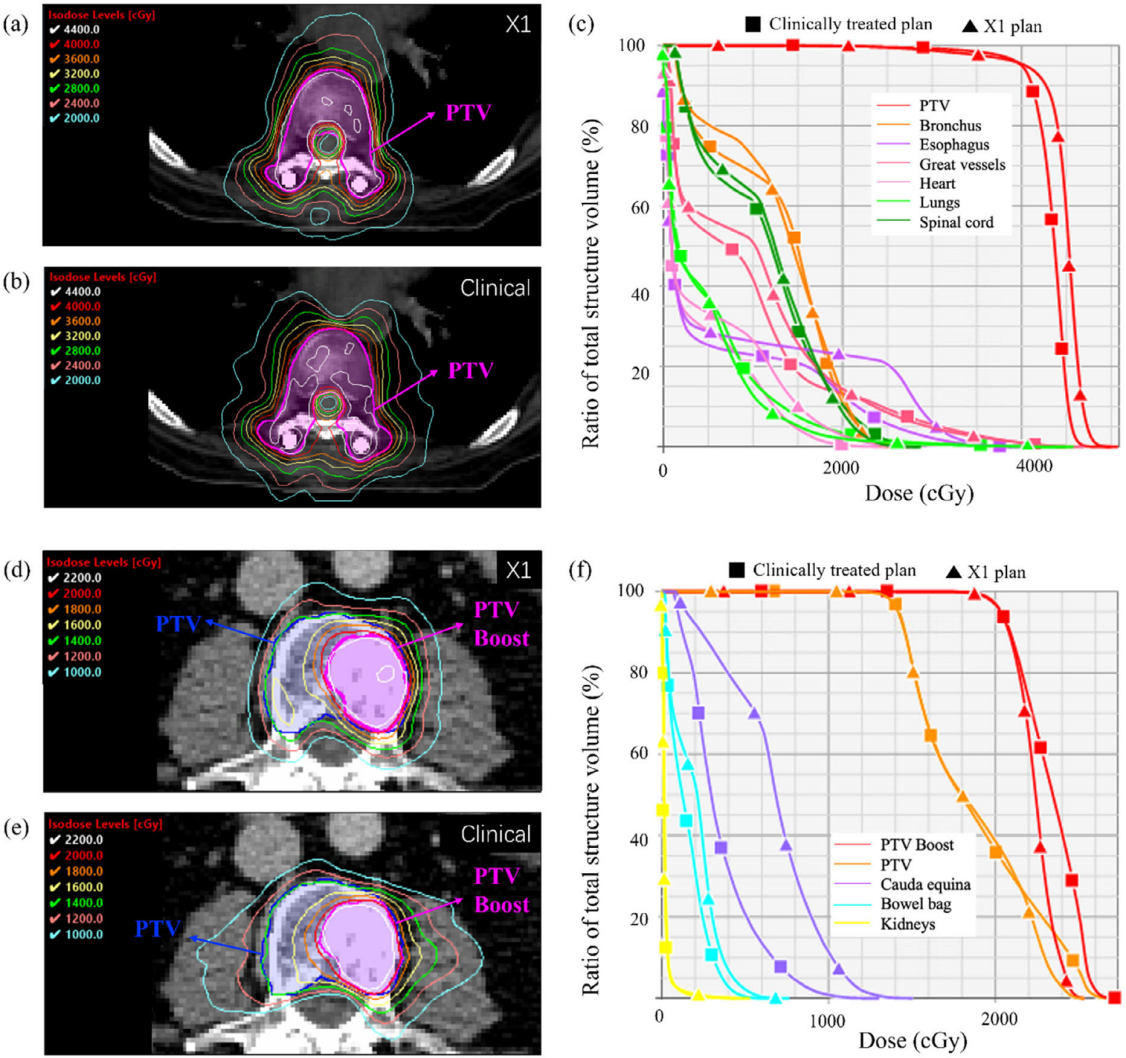
Comparisons of dose distributions and dose‐to‐volume histograms between X1 plans and clinical plans for a representative single‐dose level case(a–c) and a SIB case (d–f), respectively. Rx for single dose level is 40 Gy in five fractions. Two dose levels prescribed for SIB plans: 20 and 14 Gy, respectively, in one fraction. SIB, simultaneously integrated boost.

**FIGURE 2:**
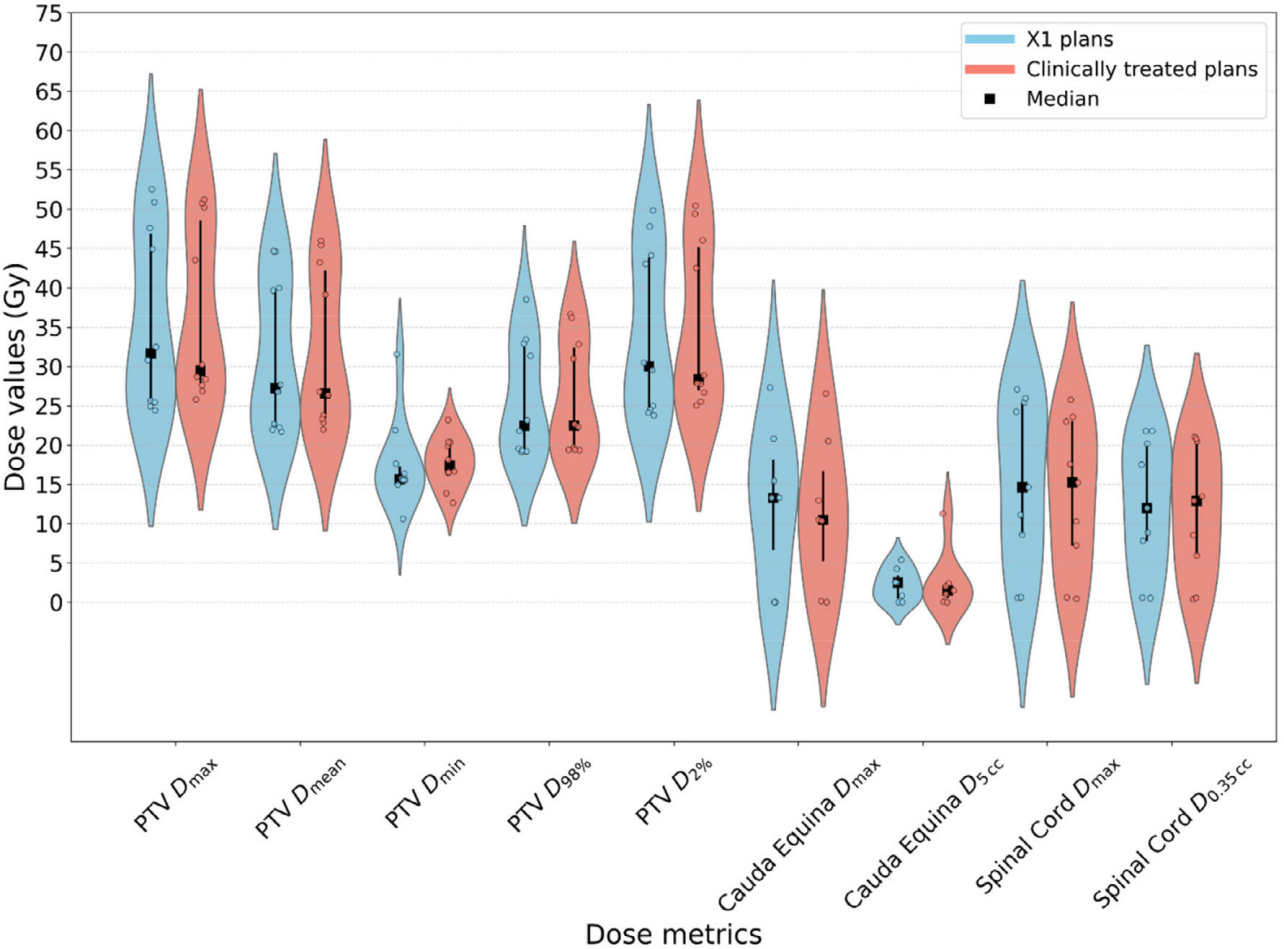
Violin plots comparing PTV, cauda equina, and spinal cord dose metrics of RefleXion X1 plans and clinically treated plans. Vertical bars represent the inter‐quartile range and black square represents median values. PTV, planning target volume.

**FIGURE 3:**
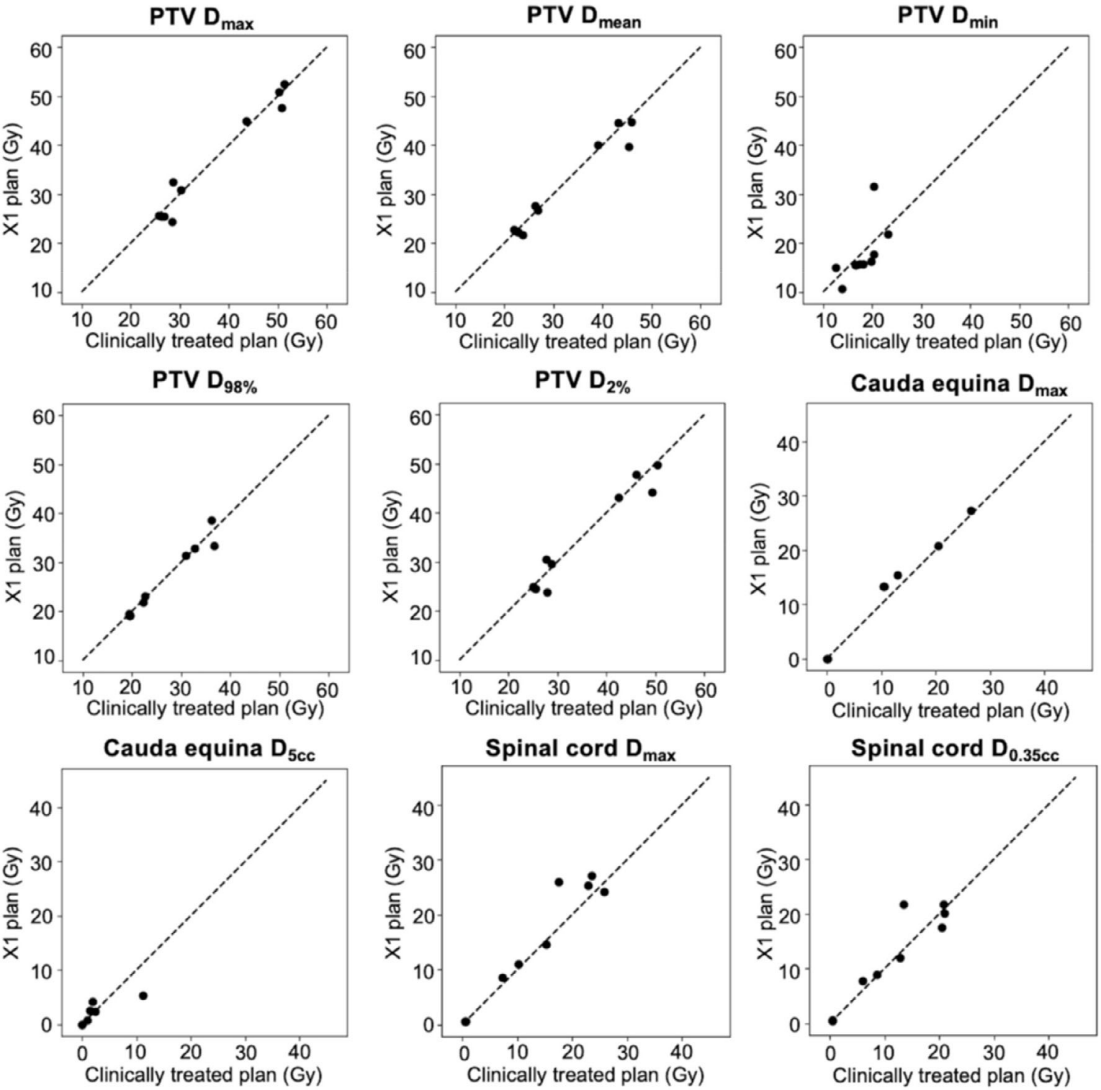
Paired comparison of dosimetric parameters for RefleXion X1 and clinical plans for spine SBRT cases. Each dot represents one patient case. SBRT, stereotactic body radiotherapy.

We successfully utilized the RefleXion X1 system to generate spine SBRT plans meeting our institutional planning guidelines. Comparable quality to that of clinically treated plans previously treated at our institution was observed. Figure 1 showcases axial dose distributions and dose‐volume histograms (DVHs) of RefleXion X1 plans and clinically treated plans generated for a representative single‐dose level case and a SIB case. The average CI values across all 10 cases were for 1.20 and 1.18, respectively for X1 plans and clinically treated plans (p=0.674), indicating comparable conformity. Table 3 summarizes the dosimetric metrics of interest for X1 and clinically treated plans. Across all PTV metrics, including $D_{98\%}$, $D_{2\%}$, $D_{\max}$, $D_{\min}$, and $D_{\mathrm{mean}}$, the average dose values and standard deviation generated by RefleXion X1 were comparable to those in clinically treated plans. The same observation can be found for main OARs, including the spinal cord and cauda equina. The *p*‐values for these metrics all exceed 0.05, indicating no statistically significant differences except slightly higher $D_{\max}$ for cauda equina (p=0.05) in X1 plans. The violin plot shown in Figure 2 provides a visual representation of the distribution of dose metrics across all plans. Figure 3 presents scatter plots showing the direct correlation between X1 plans and clinically treated plans for each dosimetric parameter, with most data points clustering near the diagonal line indicating strong agreement between the clinical and RefleXion plans.

**TABLE 3:** Summary of dosimetric parameters for RefleXion X1 plans and clinically treated SBRT plans, respectively.

		Mean Dose (min∼max) (Gy)		Dose differences (%Rx)		
Target	Metrics	X1 plans	Clinically treated plans	Mean	SD	*p*‐value
**PTV**	$D_{98\%}$	25.82 (19.10～38.54)	25.95 (19.34～36.66)	2.55	2.46	0.77
	$D_{2\%}$	34.25 (23.75～49.83)	34.89 (25.04～50.43)	6.75	6.68	0.56
	$D_{\max}$	36.01 (24.40～52.53)	36.25 (25.79～51.23)	7.10	6.30	0.70
	$D_{\min}$	17.54 (10.60～31.54)	17.81 (12.64～23.20)	10.01	7.41	0.23
	$D_{\mathrm{mean}}$	31.21 (21.69～44.67)	31.84 (21.99～45.98)	5.15	4.79	0.77
**Cauda equina**	$D_{\max}$	12.87 (0～27.32)	11.57 (0～26.54)	6.45	6.82	**0.05**
	$D_{5\mathrm{cc}}$	2.23 (0～5.41)	2.61 (0～11.27)	6.06	9.07	0.92
**Spinal cord**	$D_{\max}$	15.35 (0.56～27.07)	13.74 (0.44～25.76)	6.19	6.38	0.13
	$D_{0.35\mathrm{cc}}$	12.33 (0.52～21.78)	11.56 (0.39～21.02)	5.48	6.57	0.57

*Note*: Statistically significant *p*‐value < 0.05 is shown in bold font. Dose differences are calculated by: |$\frac{X1\;\mathrm{dose}-\mathrm{clinical}\;\mathrm{dose}}{\mathrm{Prescription}\;\mathrm{Dose}\;(\mathrm{Rx})\;}|\times 100\%$.

Moreover, comparisons of CI, HI, and GI between X1 plans and clinically treated plans were summarized in Table 4. All the evaluated metrics have similar mean and standard deviation values and no statistically significant differences, demonstrating comparable performance of X1 plans compared to clinically treated plans.

**TABLE 4:** Comparison of conformity index (CI), homogeneity index (HI), and gradient index (GI) between X1 plans and clinically treated plans.

Metrics	Clinically treated plans	X1 plans	*p*‐value
**CI**	1.18±0.14	1.19±0.11	0.674
**HI**	0.63±0.17	0.65±0.17	0.820
**GI**	1.06±0.03	1.05±0.00	0.285

## 4 DISCUSSION

We investigated the feasibility of using the RefleXion X1 system spinal SBRT. Compared to clinically treated plans, minimal statistically significant differences in key PTV metrics such as $D_{\max}$ (p=0.70), $D_{\mathrm{mean}}$ (p=0.77), and $D_{\min}$ (p=0.23) were observed alongside with comparable CI (p=0.67), HI (p=0.82), and GI (p=0.29) values. Although X1 plans demonstrated slightly higher average maximum doses to the cauda equina (X1 plans: 12.87 Gy, clinically treated plans: 11.57 Gy, p=0.05), the doses remained within institutional dose constraints. This slight discrepancy could be attributed to differences in dose calculation algorithms, as X1 plans were computed using CCCS algorithm while clinical plans employed AXB algorithm. The CCCS uses a simplified convolution approach for heterogeneity corrections and may show slightly degraded dose calculation accuracy in tissue interfaces.^19^ The AXB algorithm explicitly solves linear Boltzmann transport equation, which often provides enhanced accuracy for dose calculations in heterogeneous mediums, particularly near bone‐tissue interfaces compared to convolution‐based algorithms. These differences can result in small variations in calculated doses, particularly in regions with significant density variations.^19^, ^20^

Treatment delivery considerations also merit discussion when comparing the X1 system to conventional approaches. Due to its slice‐by‐slice treatment delivery approach, the RefleXion X1 system requires significantly longer treatment times compared to conventional VMAT delivery. The estimated average treatment time for RefleXion X1 plans was ∼30 min, compared to the typical 5–10 min for spine SBRT delivery on conventional C‐arm linear accelerators using VMAT. This extended treatment time results from the X1's slice‐by‐slice delivery technique with four‐pass delivery and discrete couch movement between beam stations. This difference represents a practical consideration for clinical workflow and patient throughput.

Another important point to clarify is that the RefleXion X1 system can deliver radiation using either single‐pass or four‐pass modes, with the latter one intended for more complex treatments like SBRT and biology‐guided radiotherapy (BgRT).^7^, ^12^ All the results reported in the study are based on four‐pass plans following this recommendation. In fact, in our study, we found no significant differences in plan quality between single‐pass and four‐pass plans for spine SBRT cases. However, it is important to acknowledge that this comparable performance observed may not be generalizable to other treatment sites.

Several limitations of this study should be acknowledged. First, this investigation included a relatively small sample size of 10 cases, which may not be sufficient to fully capture the variability in treatment plan optimization across the diverse range of spinal anatomy and tumor presentations encountered in clinical practice. While our selected cases encompassed different anatomical locations and planning complexities, a larger‐scale study would provide more robust statistical power and broader generalizability of our findings. Future studies should include a larger cohort to validate these preliminary results and establish more definitive conclusions regarding the clinical implementation of the RefleXion X1 system for spinal SBRT. While this study focused on the feasibility of treatment planning using the X1 system, its BgRT mode leveraging PET imaging for intra‐treatment tumor tracking in spine SBRT remains uninvestigated. As a cutting‐edge treatment modality, BgRT treatment has the potential to realize functional‐guided therapy based on tumor response.^21^ Further investigations should explore the feasibility of applying BgRT to treat spine tumors.

## 5 CONCLUSION

This study evaluated the feasibility and dosimetric quality of planning spinal SBRT using RefleXion X1 system, demonstrating its capability in producing high‐quality treatment plans to meet all institutional planning guidelines. Comparisons with clinical plans from other treatment platforms revealed no significant differences in most dosimetric parameters of interest.

## AUTHOR CONTRIBUTIONS

Haozhao Zhang, Shunyu Yan, and Chenyang Shen designed the study. Haozhao Zhang, Shunyu Yan, and Chenyang Shen collected and analyzed data. Chenyang Shen, Andrew Godley, Thomas Banks, Rameshwar Prasad, Yang Kyun Park and Bin Cai provided clinical supervision and input to the study. Haozhao Zhang, Shunyu Yan, and Chenyang Shen drafted the manuscript, and all authors revised and approved the final manuscript.

## CONFLICT OF INTEREST STATEMENT

The authors declare no conflicts of interest.
